# Continuous attractor dynamics in spatial navigation: from population geometry to flexible computation

**DOI:** 10.3389/fnins.2026.1788255

**Published:** 2026-04-08

**Authors:** Yani Chen, Mu Hua, Xuelong Sun, Jigen Peng

**Affiliations:** 1 Machine Life and Intelligence Research Center, Guangzhou University, Guangzhou, China; 2 School of Mathematics and Information Science, Guangzhou University, Guangzhou, China; 3 School of Computing and Mathematical Sciences, University of Leicester, Leicester, United Kingdom

**Keywords:** continuous attractor networks, grid cells, head-direction cells, neural field equations, pattern formation

## Abstract

A central computational problem in spatial navigation is how spatial representations remain stable under noise and uncertainty, and update reliable estimations of continuous variables such as head-direction and position, which respectively rely on the head-direction system and the grid-cells system in the entorhinal cortex. The two systems demonstrate strong population-level dynamics, suggesting a potential framework to explain the critical problem of spatial representations. Currently, the framework involves continuous attractor networks and the neural field theories as an unified perspective, from which the population activity can be described as evolving of continuous variables on a low-dimensional attractor manifold, together with the selective instantiation of these dynamics across symmetry-related or context-dependent subspaces. From this viewpoint, a key question is how different sources of information, such as self-motion, sensory cues and environmental structure, interact with attractor dynamics to regulate the evolution and stability of population states. Specifically, external inputs can stabilize attractor states by anchoring them to landmarks; intrinsic network connectivity, symmetry, and multi-timescale dynamics determine whether an attractor is stable and whether it supports continuous motion; environmental boundaries and geometric constraints can systematically shape the local geometry of spatial activity patterns; direction- or context-dependent signals may selectively recruit neuronal subpopulations with specific tuning preferences; and cross-level organization of attractor dynamics, enabling a unified representational and control framework from individual decision-making to collective behavioral organization. Through the joint action of these mechanistic dimensions, continuous attractor representations are able to support the core computations required for navigation. More broadly, this perspective provides a theoretical foundation for understanding how continuous spatial representations are computed, read out, and flexibly manipulated to support planning and behavioral control.

## 1 Introduction

A central computational problem in spatial navigation is how spatial representations maintain stability under noise and uncertainty, and update reliable estimates of continuous variables such as direction and position (McNaughton et al., 2006; Khona and Fiete, 2022; Seelig and Jayaraman, 2015). A number of experimental evidence indicate that this problem may solved through population-level dynamics across species and brain regions. For example, hippocampal place-cell population fires selectively for spatial locations (O'Keefe and Dostrovsky, 1971; O'Keefe, 1976); entorhinal grid-cell population exhibits periodic hexagonal firing fields in two-dimensional environments (Hafting et al., 2005; Moser et al., 2008, 2014); head-direction population in both insects and rodents displays stable directional coding without visual cues (Turner-Evans et al., 2017; Kim et al., 2017) together with the ability to update this code using self-motion information (Taube et al., 1990; Seelig and Jayaraman, 2015; Angelaki and Laurens, 2020). Together, these findings raise a shared theoretical question: how can the nervous system support population dynamics to remain stable under noise and uncertainty and smooth update during movement?

Continuous attractor neural networks (CANNs) and neural-field equations provide a natural theoretical framework for addressing this question. Through recurrent connectivity with local excitation and long-range inhibition, neural populations can form stable activity bumps (a localized, self-sustained peak of population activity) or spatially periodic patterns whose steady states are organized as low-dimensional attractor manifolds, such as a ring for heading direction or a two-dimensional torus for spatial position, along which network states can evolve continuously without altering their overall shape (Zhang, 1996; Fung et al., 2010; Wu et al., 2016; Burak and Fiete, 2009; Bressloff, 2011). This class of dynamics naturally supports the encoding of continuous variables and their self-motion-driven updating. Recent population recordings and geometric analyses further show that grid-cell activity can indeed occupy a low-dimensional state-space manifold with toroidal topology (Gardner et al., 2022), and that grid-cell dynamics exhibit signatures of continuous attractor behavior (Yoon et al., 2013), reinforcing the attractor-manifold view as a unifying representational language (Khona and Fiete, 2022).

Crucially, however, spatial representations in biological systems are influenced by many factors. Experimental observations reveal multiple distinct forms of variability in spatial representations. Under conditions dominated by self-motion input, head-direction can exhibit gradual drift (Taube et al., 1990; Seelig and Jayaraman, 2015); external sensory cues can anchor or recalibrate these representations (Fyhn et al., 2007; Harootonian et al., 2022); environmental boundaries can induce deformation or shearing of grid patterns (Stensola et al., 2015; Hafting et al., 2005); finally, spatial representations can switch discretely across compartments or contexts (Derdikman et al., 2009; Waaga et al., 2022). From a dynamical-systems perspective, these diverse phenomena point to a small set of fundamental degrees of freedom, through which biological circuits shape attractor dynamics (Zhang, 1996; Amari, 1977; Burak and Fiete, 2009; Wu et al., 2016; Khona and Fiete, 2022). In particular, four classes of mechanisms emerge across theoretical models and experimental systems: (i) external inputs that drive or bias population activity, such as landmark signals (Knierim et al., 2014; Jeffery et al., 2016; Kutschireiter et al., 2023; Gu et al., 2008; Chen et al., 2024); (ii) intrinsic network structure plays a central role in shaping attractor dynamics. Recurrent connectivity with local excitation and longer-range inhibition can generate and stabilize bump-like activity patterns (Zhang, 1996; Burak and Fiete, 2009), introducing asymmetry into the connectivity can convert stationary bumps into drifting solutions, supporting angular path integration (Song and Wang, 2005; Fung et al., 2010), and multi-timescale synaptic processes, such as depression or adaptation, can further modulate stability and induce traveling waves or state transitions (Kilpatrick and Bressloff, 2010a; Faye, 2013; Chen et al., 2024); (iii) boundary and domain constraints imposed by environmental geometry, which reshape the attractor landscape (Monsalve-Mercado and Leibold, 2020; López et al., 2024); (iv) selection among multiple attractors, enabling realignment, remapping, and context-dependent reference-frame choice (Derdikman et al., 2009; Fyhn et al., 2007; Skaggs and McNaughton, 1998; Grieves et al., 2016); and (v) cross-level coupling from representation to behavior, whereby attractor state variables, when linked to motor output, organize control at the individual level and can scale to collective order through coupling between internal states (Salahshour and Couzin, 2025) . How these degrees of freedom interact to support integration, calibration, and flexible navigation remains a central open problem.

In this Review, we adopt a unified continuous-attractor and neural-field perspective to organize experimental and theoretical results around these core dynamical degrees of freedom (Wilson and Cowan, 1972; Amari, 1977; Bressloff, 2011; Wu et al., 2016). We argue that phenomena in head-direction and grid-cell systems can be understood as different manifestations of how external drive, intrinsic structure, boundary constraints, and attractor selection jointly shape population dynamics (Taube et al., 1990; Seelig and Jayaraman, 2015; Hafting et al., 2005; Gardner et al., 2022). By framing spatial representation as controlled dynamics on low-dimensional manifolds, this perspective provides a principled route toward linking representational geometry, circuit mechanisms, and downstream navigational computation.

## 2 Experimental phenomena of continuous spatial representations

Before turning to formal models, we first review key experimental phenomena that motivate an attractor-based dynamical view of spatial representation. Across species and spatial scales, head-direction and grid systems exhibit stable population activity patterns that can drift, distort, realign, or switch under different sensory and environmental conditions, suggesting that spatial representations are shaped by multiple interacting mechanisms.

### 2.1 The head-direction system

**Figure 1:**
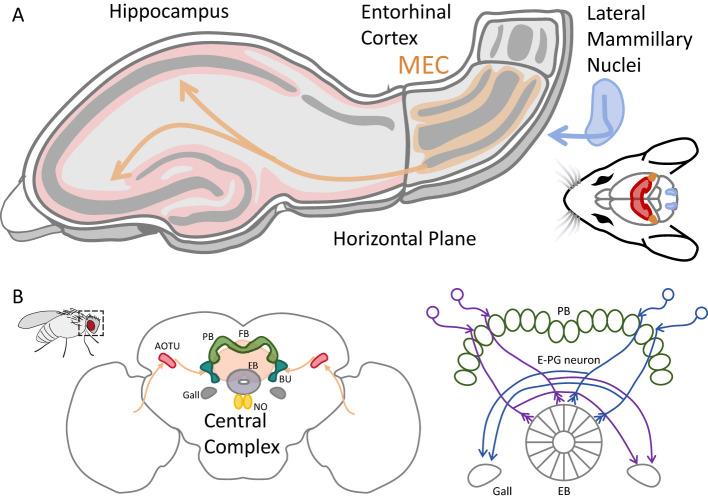
Anatomical substrates of navigation systems in rodents and Drosophila. **(A)** Schematic organization of the entorhinal-hippocampal circuit in rodents. Head-direction cells are located in the lateral mammillary nuclei (LMN) and related thalamic structures; grid cells and border cells reside in the medial entorhinal cortex (MEC); place cells are found in the hippocampus. This anatomical overview highlights the connected architecture that supports population-level spatial representations. **(B)** Organization of the insect central complex (adapted from (Turner-Evans et al., 2020)), including the anterior optic tubercle (AOTU), bulb (BU), ellipsoid body (EB), protocerebral bridge (PB), noduli (NO), fan-shaped body (FB), and associated structures. The right panel illustrates E-PG neurons, which form a ring-like population encoding heading orientation. Together, these systems provide circuit motifs capable of supporting continuous population dynamics underlying spatial orientation.

Figure 1 summarizes the anatomical substrates of navigation circuits in mammals and insects, providing the structural context for the population-dynamical principles discussed below. In the Drosophila central complex (CX) (Figure 1B), individual E-PG neurons are spatially tuned to specific angular positions, with their activity peaks shifting smoothly with self-motion as the animal turns (Seelig and Jayaraman, 2015; Kim et al., 2017; Turner-Evans et al., 2017; Kakaria and De Bivort, 2017; Turner-Evans and Jayaraman, 2016; Turner-Evans et al., 2020). Closely related compass-like heading representations have also been reported in other insects, such as monarch butterflies, and in vertebrates including rodents (Homberg, 2004; Beetz et al., 2022; Stone et al., 2017). When visual gain is manipulated or visual-self-motion inconsistency is introduced, the activity bump may partially track, jump, or become unstable (Seelig and Jayaraman, 2015; Kim et al., 2019; Fisher et al., 2019), suggesting that head-direction representations are not simple linear filters but may instead reflect continuous attractor dynamics (dynamics in which a family of equivalent steady states forms a continuous manifold in population activity space).

**Figure 2:**
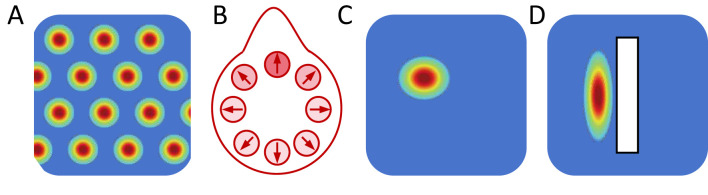
Representative firing fields of single spatially tuned neurons. Example firing patterns of **(A)** a grid cell, showing multiple periodic firing fields arranged in a hexagonal lattice across physical space; **(B)** a head-direction cell, exhibiting a unimodal directional tuning curve as a function of the animal's heading; **(C)** a place cell, with a localized firing field in a specific spatial location; and **(D)** a boundary cell, activated when the animal is near environmental borders. These single-cell firing patterns constitute the empirical basis for population-level models, where ensembles of such neurons give rise to low-dimensional attractor manifolds.

In rodents, the head-direction network is tightly coupled to vestibular-mammillary pathways (Figure 1A) and supports angular-velocity-driven integration (Taube et al., 1990; Goodridge and Taube, 1997; Yoder and Taube, 2014). Individual cells (Figure 2) show persistent activity and stable directional tuning (Yoshida and Hasselmo, 2009; Angelaki and Laurens, 2020). This is functionally isomorphic to insect systems, an intrinsic attractor provides a stable compass, self-motion inputs update it continuously, and external landmarks correct drift and anchor the internal representation to the external reference frame (Seelig and Jayaraman, 2015; Knight et al., 2014; Jeffery et al., 2016).

### 2.2 The grid-cell system

Another class of navigational cells in entorhinal-hippocampal structure (Figure 1A) are grid cells (Figure 2), which are characterized by hexagonally periodic firing in two-dimensional space (Hafting et al., 2005; Fyhn et al., 2004; Moser et al., 2008). Subsequent studies show that grid scale is discretised into modular levels (Stensola et al., 2012), and that grid maps can realign and remap under environmental manipulations (Fyhn et al., 2007; Waaga et al., 2022). Grid representations are not rigid crystals, environmental boundaries and geometry can distort and reorganize grid patterns (Monsalve-Mercado and Leibold, 2020), and more general boundary conditions and diffusion/propagation mechanisms can systematically influence the structure and stability of Turing patterns (López et al., 2024). In addition, grid periodicity is coupled to network state, when theta oscillations are reduced, grid spatial periodicity may be disrupted (Brandon et al., 2011; Koenig et al., 2011), and the absence of visual input can strongly impair grid firing (Chen et al., 2016). These findings suggest that grid patterns may correspond to spatially periodic attractor states whose geometry is influenced by domain boundaries and external inputs.

### 2.3 Low-dimensional geometry of population representations

Beyond single-neuron firing patterns, the geometry of population dynamics has become a key line of evidence for attractor theories. In the entorhinal cortex, grid-cell population activity has been shown to occupy a toroidal structure in state space (Gardner et al., 2022), directly revealing a low-dimensional manifold on which population activity evolves continuously. Complementing this, earlier work demonstrated signatures of low-dimensional continuous attractor dynamics in grid cells during navigation (Yoon et al., 2013). In contrast to these smoothly evolving representations, discrete attractor dynamics have been identified in cortical circuits supporting persistent activity, where population states remain stable (Inagaki et al., 2019).

A key computational implication of low-dimensional attractor geometry is that it naturally separates different classes of perturbations. Perturbations that primarily displace activity along the attractor manifold manifest as phase shifts, drift, or realignment, while preserving the global structure of the population activity pattern (Wu et al., 2016; Fung et al., 2010). By contrast, perturbations that alter pattern shape, amplitude, or stability effectively push activity away from the manifold, resulting in deformation or destabilization of the representation. Operationally, this distinction can be identified by examining whether population activity preserves its global structure while undergoing a phase shift, or instead exhibits systematic changes in its geometry.

## 3 Representational geometry and dynamical degrees of freedom in continuous attractor manifolds

Continuous attractor networks and neural-field theory are often viewed as coming from different traditions: Continuous attractor network models were initially developed to explain head-direction coding and path integration (Skaggs et al., 1994; Samsonovich and McNaughton, 1997; Zhang, 1996); subsequent work extended these models to incorporate spiking dynamics and circuit-level implementations (Xie et al., 2002; Song and Wang, 2005) and more recent studies have linked such attractor dynamics to biologically realistic recurrent inhibitory circuitry in the entorhinal cortex (Burak and Fiete, 2009; Couey et al., 2013). The latter is widely used for mathematical analyses of cortical spatial pattern formation (Bressloff, 2011; Coombes, 2005). However, from a dynamical-systems perspective, they describe the same class of continuous population-dynamical mechanisms at different description levels (Wu et al., 2016; Ermentrout and Terman, 2010; Khona and Fiete, 2022). Unifying them within a single framework enables a systematic understanding of the continuous spatial representations manifested by head-direction and grid cells at both geometric and dynamical levels (Angelaki and Laurens, 2020; Moser et al., 2014).

**Figure 3:**
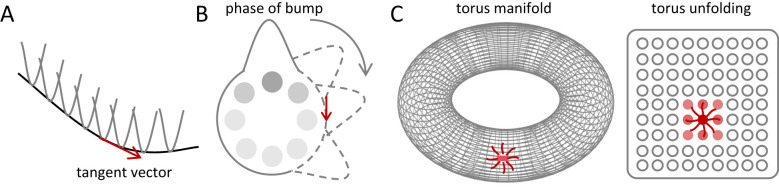
Geometric structure of continuous attractor manifolds. Continuous attractor networks generate low-dimensional manifolds of equivalent steady states, along which population activity can move smoothly while preserving its overall pattern. The manifold structure naturally separates perturbations into two classes: (i) tangent directions, which shift activity along the manifold without restoring forces, and (ii) transverse (normal) directions, along which perturbations are suppressed or amplified, leading to stabilization or deformation of the pattern. **(A)** Schematic illustration of a one-dimensional attractor manifold. The curved cross-section represents local stability in the transverse direction, whereas the tangent direction corresponds to neutrally stable displacements. **(B)** Ring manifold corresponding to head-direction coding. The position of the activity bump along the ring encodes the represented angle; a displacement along the ring corresponds to a phase shift. **(C)** Toroidal manifold corresponding to grid-cell populations. The red region illustrates the local recurrent connectivity of a representative neuron within the two-dimensional periodic population pattern.

In continuous attractor networks, recurrent connectivity with local excitation and long-range inhibition gives rise to stable population activity patterns whose steady states are not isolated fixed points, but instead form a continuous family parameterised by one or more continuous variables. Collectively, these equivalent states constitute a low-dimensional attractor manifold (Figure 3), for example, a ring manifold encoding heading direction, along which network states can move continuously without disrupting the global activity profile (Zhang, 1996; Fung et al., 2010; Wu et al., 2016). This manifold structure provides a natural substrate for representing continuous variables at the population level. From a data-analytic perspective, the notion of a low-dimensional attractor manifold can be made concrete by considering the population activity vector across simultaneously recorded neurons. Dimensionality-reduction or manifold-learning techniques (e.g., PCA) can then be used to visualize the dominant structure of this activity.

Neural-field theory offers a more general mathematical formulation of this principle. In neural-field models (Equation 1), population activity is treated as a continuous field evolving under recurrent coupling and nonlinear activation (Wilson and Cowan, 1972, 1973; Amari, 1977; Ermentrout, 1998; Bressloff, 2011). Specifically, *u*(*x, t*) denotes the population activity at position *x* in the represented feature space (e.g., angular direction or spatial location), ω(*x, x*′) is the synaptic connectivity kernel describing how activity at *x*′ influences activity at *x*, and *F*(·) is a nonlinear activation function that converts activity into effective firing rates. The time constant τ sets the intrinsic timescale of the population dynamics. Such a model can generate stable activity bumps, stripe patterns, or periodic lattice solutions (Figure 3) (Amari, 1977; Coombes, 2005; Bressloff et al., 2001). Similarly, when neural-field equations possess translational or rotational invariance, a single solution generates a continuous family of equivalent solutions through the action of the symmetry group. Geometrically, this family forms a low-dimensional manifold in population-activity space. Dynamically, if the linearized operator has zero eigenvalues associated with the symmetry directions, while all remaining eigenvalues have negative real parts, then this manifold constitutes a low-dimensional attractor in the dynamical-systems sense, closely aligning neural-field steady states with the concept of continuous attractors (Coombes, 2005; Bressloff, 2011).


$$\begin{array}{l} \tau \frac{\partial u\left(x,t\right)}{\partial t}=-u\left(x,t\right)+\int \omega \left(x,x^{\prime }\right)F\left(u\left(x^{\prime },t\right)\right)dx^{\prime }. \end{array}$$ (1)

Within this unified neural-field/continuous-attractor framework, a natural distinction between navigation-related representations may arise from the dimensionality and symmetry of the represented variable itself. From this perspective, the differences between head-direction and grid-cell systems can be understood as consequences of distinct geometries of the underlying attractor manifolds. In head-direction systems, neural activity is defined on a one-dimensional periodic domain. Local-excitation/long-range-inhibition connectivity supports a single stable activity bump, and the family of equivalent steady states forms a one-dimensional ring manifold (Zhang, 1996). In contrast, in two-dimensional space, neural-field models possessing Euclidean symmetry can generate periodic spatial patterns via Turing-type instabilities (Ermentrout and Cowan, 1979; Ermentrout, 1998; Bressloff et al., 2001). Turing-type instabilities, in which a spatially uniform state becomes unstable to activity patterns with a characteristic wavelength, can give rise to periodic spatial patterns. For example, in two-dimensional neural fields with specific recurrent connectivity, such mechanism naturally selects hexagonal arrangements (Figure 2), closely resembling the regular firing fields observed in grid cells (Fuhs and Touretzky, 2006; Couey et al., 2013; Burak and Fiete, 2009). Population-level recordings revealing toroidal state-space structure provide direct empirical support for this geometric interpretation (Yoon et al., 2013; Gardner et al., 2022; Guanella et al., 2007).

## 4 From spatial patterns to navigation-related computations in attractor networks

While attractor geometry constrains the space of possible representations, navigation depends on how population activity is driven, stabilized, and reorganized on these low-dimensional manifolds by self-motion and environmental signals. During navigation, neural populations must integrate internal motion-related inputs, incorporate external sensory information such as landmarks and boundaries, and flexibly adapt representations across contexts. Functionally, this requires three core operations: (i) continuous state updating along the attractor manifold driven by self-motion signals; (ii) correction and calibration of accumulated error through interactions with environmental structure and sensory cues; and (iii) context-dependent selection or reorganization of reference frames and spatial representations (selection and remapping). From this perspective, phenomena such as drift, boundary-induced distortion, and cross-environment remapping reflect how attractor dynamics are modulated by external inputs, environmental geometry, and internal network structure, thereby revealing both the computational capabilities and the inherent constraints of navigational circuits.

### 4.1 External signals as extrinsic forcing of attractor dynamics

**Figure 4:**
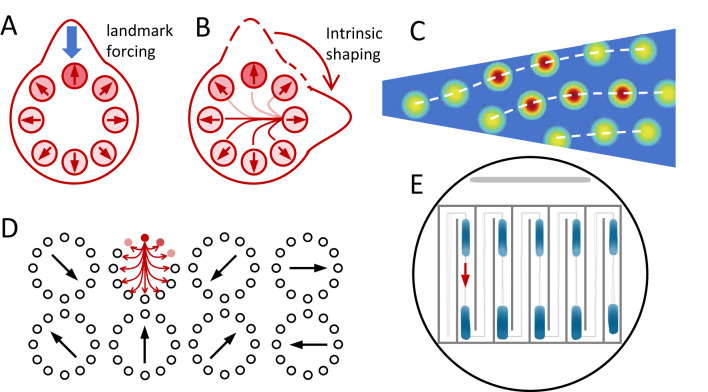
Multiple classes of dynamical operations in navigation circuits (Table 1). Continuous attractor dynamics can be modulated through distinct mechanisms that affect how population states evolve on manifolds. **(A)** External anchoring: Landmark inputs shift the effective attractor (and its basin) in state space, relocating the stable bump position and pulling activity toward an externally defined reference. **(B)** Structural modulation: Changes in recurrent connectivity, or synaptic dynamics reshape the local flow field on the manifold, enabling drifting solutions. **(C)** Boundary and domain constraints: Environmental geometry alters the effective domain of the attractor, leading to deformation or shear of spatial firing patterns. **(D)** Attractor selection: When multiple symmetry-related attractor subspaces exist, contextual or directional signals bias activity toward one subspace, implementing input-dependent indexing rather than structural reconfiguration. **(E)** Example of grid-cell firing fields resulting from selective instantiation of one attractor subspace.

External signals can influence continuous attractor dynamics in qualitatively distinct ways, depending on how they enter the network equations. Figure 4 and Table 1 should be read as a classification scheme. One prominent class consists of inputs that act as extrinsic forcing terms (Figure 4A), directly biasing population activity without modifying the underlying recurrent connectivity. In neural-field and continuous-attractor formulations, such inputs enter additively and reshape the effective potential landscape on which population states evolve (Chen et al., 2024).

**Table 1:** Mechanism classes linking phenomena to attractor dynamics.

Mechanism class	Representative phenomena	Key references
Extrinsic forcing	Landmark anchoring, cue conflict resolution, et al.	Jeffery et al., 2016; Kutschireiter et al., 2023; Gu et al., 2008; Chen et al., 2024
Intrinsic structural modulation	Velocity-driven drift, traveling bumps, et al.	Zhang, 1996; Song and Wang, 2005; Fung et al., 2010; Burak and Fiete, 2009
Boundary and domain constraints	Grid-field distortion near walls, shearing asymmetry, et al.	Monsalve-Mercado and Leibold, 2020; López et al., 2024
Attractor selection and multistability	Grid fragmentation, place-field repetition, et al.	Derdikman et al., 2009; Fyhn et al., 2007; Skaggs and McNaughton, 1998; Grieves et al., 2016; Spiers et al., 2015
Cross-level organization	Collective behavioral control via internal attractor states	Salahshour and Couzin, 2025

In navigation circuits, landmark-related sensory cues are natural candidates for this form of extrinsic forcing. By providing spatially or directionally selective input, landmarks can stabilize the position of an activity bump or constrain the phase of a grid pattern, effectively anchoring an otherwise drifting attractor to an external reference frame. Importantly, in this regime the geometry of the attractor manifold itself, such as a ring for head direction or a torus for grid representations, remains intact. External inputs primarily determine where on the manifold the system resides, rather than how the manifold is structured.

From a computational perspective, extrinsic forcing implements calibration and error correction. When multiple cues are present, their relative strengths determine the location of the stable state on the attractor, naturally giving rise to weighted cue integration. Under conflict, changes in the forcing terms can shift the balance between competing reference frames without requiring any reconfiguration of the internal network architecture (Chen et al., 2024). Thus, extrinsic inputs regulate navigational direction by biasing state within a fixed representational geometry.

### 4.2 Structural modulation and intrinsic shaping of attractor dynamics

A fundamentally different role of external signals emerges when they modulate the effective recurrent structure of the network rather than acting as additive drives. In this case, inputs influence navigation by altering intrinsic coupling properties, such as symmetry, gain, or time-scale separation, thereby reshaping the attractor dynamics themselves (Figure 4B). Velocity-related signals often operate in this regime: instead of simply pushing the system along an existing attractor, they modify the recurrent interactions so that previously static patterns become capable of systematic motion (Zhang, 1996; Burak and Fiete, 2009).

In continuous attractor and neural-field models, such structural modulation is commonly implemented through asymmetric connection, synaptic dynamics, or slow adaptive processes (Zhang, 1996; Tsodyks and Markram, 1997; York and van Rossum, 2009; Kilpatrick and Bressloff, 2010a,b; Faye, 2013). These mechanisms can convert stationary bumps into drifting solutions, generate traveling waves, or induce pattern deformation even in the absence of explicit external forcing. From this viewpoint, self-motion does not merely drive the system forward along a representational manifold, it actively reconfigures the local flow on that manifold, determining whether motion is possible and how it unfolds.

More generally, intrinsic network structure defines the computational regime in which a navigation circuit operates. Depending on connectivity and synaptic dynamics, the same network may support stable maintenance, continuous updating, or rapid transitions between states. Such regime flexibility provides a natural mechanism for balancing robustness and adaptability under changing behavioral demands and noise conditions (Braun and Mattia, 2010; Gigante et al., 2009). Crucially, because these effects arise from modifications to the effective kernel or coupling structure, they may link connectivity geometry to the stability of the attractor manifold itself.

### 4.3 Boundary and domain constraints on attractor dynamics

Environmental geometry introduces an additional class of constraints by shaping the domain on which attractor dynamics unfold. Both theoretical analyses and experimental observations demonstrate that boundaries can distort grid-cell firing patterns, induce reorganization, and alter local metric properties (Figure 4C) (Monsalve-Mercado and Leibold, 2020). More generally, boundary conditions and diffusion or propagation properties systematically affect the stability and geometry of Turing-type patterns in neural fields (López et al., 2024; Monsalve-Mercado and Leibold, 2020).

From a computational perspective, boundaries act as structured priors imposed on path-integration representations. They restrict unconstrained drift, shape the effective geometry of the representational space, and provide long-term constraints that limit error accumulation (McNaughton et al., 2006; Khona and Fiete, 2022). Accordingly, sensitivity of grid representations to environmental geometry can be understood as an inevitable consequence of solving continuous field equations on finite domains: environmental structure enters the dynamics through boundary conditions, reshaping the set of accessible attractor states. Shearing-induced asymmetries further illustrate how geometric constraints can systematically bias spatial representations in experimentally testable ways (Stensola et al., 2015).

### 4.4 Selection among attractors as a mechanism for realignment and remapping

Navigation across different contexts requires not only continuous updating and calibration, but also mechanisms for flexibly instantiating distinct spatial representations. Empirically, grid-cell activity can undergo realignment when animals transition between connected environments (Carpenter et al., 2015); in multicompartment settings, grid representations may fragment across compartments (Derdikman et al., 2009); hippocampal place-cell representations can exhibit repetition or global remapping across environments (Skaggs and McNaughton, 1998; Grieves et al., 2016; Spiers et al., 2015). Lesion studies further indicate that representational stability and remapping are distributed across interacting circuits rather than controlled by a single pathway (Schlesiger et al., 2018; Hales et al., 2014; Harland et al., 2017), highlighting the importance of coordinated dynamics across head-direction, entorhinal, and hippocampal systems (Save and Sargolini, 2017).

From an attractor perspective, these phenomena can be decomposed into three related operations. Realignment corresponds to a global rotation or translation of an existing attractor manifold to match a new reference frame, without altering its internal geometry. When network architecture supports multiple structurally equivalent attractor subspaces, selection can be implemented through input-dependent gating or indexing that determines where continuous attractor dynamics are instantiated (Figures 4D, E). Directional or contextual signals bias activity toward a particular symmetry-related subspace, giving rise to spatial representation fragmentation without requiring changes in intrinsic attractor dynamics.

More profound reconfiguration, involving transitions between qualitatively distinct pattern families, requires effective changes in connectivity, gain, or symmetry properties of the network (Amari, 1977; Bressloff, 2011; Agmon and Burak, 2020). While such reorganization may be necessary in some circumstances, it represents a stronger intervention at the level of network structure and is not required to account for many experimentally observed forms of remapping and context dependence.

This decomposition links cell-level remapping phenomena to network-level symmetry breaking, multistability, and parameter-dependent changes in attractor structure. Framing remapping in these terms allows the use of dynamical concepts such as basins of attraction, symmetry-related subspaces, and bifurcation structure to characterize when realignment, input-dependent selection, or genuine reconfiguration becomes necessary (Coombes, 2005; Ermentrout and Terman, 2010; Khona and Fiete, 2022).

### 4.5 Cross-level organization of attractor dynamics

Continuous attractor dynamics not only constitute a low-dimensional representational structure, but can also serve as a computational architecture for organizing behavior across levels of organization. At the level of the individual, state variables defined on an attractor manifold have been widely used to encode continuous variables such as heading direction, supported by theoretical (Zhang, 1996) and experimental evidence (Seelig and Jayaraman, 2015; Kim et al., 2017). Crucially, this state variable is not merely an internal code: when link to motor output, it can directly participate in behavioral control, allowing directional representations to translate naturally into movement decisions (Sun et al., 2020). When this structure is extended to multi-agent systems, each individual continues to maintain its own internal attractor state while receiving relative position and orientation inputs from the environment and from other agents. These inputs can act effectively on the individual's internal attractor dynamics, such that cross-individual coupling is realized through the evolution of internal states rather than through explicit representation of others. At the collective scale, this mechanism can give rise to ordered behaviors such as goal-directed motion and directional alignment (Salahshour and Couzin, 2025). From this cross-level perspective, the attractor manifold provides not only a unified representational language but also a scalable dynamical organizing principle. Phase evolution at the level of the individual and order formation at the level of the group are governed by shared constraints of stability. Continuous attractor dynamics may therefore be understood as a computational framework that retains formal consistency across organizational scales, enabling internal spatial representations to extend naturally into individual decision-making and the emergence of collective behavior.

### 4.6 Positioning continuous attractor accounts among alternative frameworks

In this section, we emphasize that the continuous attractor/neural field framework provides a dynamical language rather than a unique explanation for all observed phenomena. For example, in the head-direction system, continuous attractor models have been widely used to account for representational stability and path integration (Zhang, 1996; Xie et al., 2002; Song and Wang, 2005; Samsonovich and McNaughton, 1997), yet alternative approaches formulate heading estimation as a problem of Bayesian cue integration (Jeffery et al., 2016; Kutschireiter et al., 2023; Lakshminarasimhan et al., 2018). Similarly, in the grid-cell system, attractor models (Fuhs and Touretzky, 2006; Guanella et al., 2007; Burak and Fiete, 2009; Couey et al., 2013) coexist with oscillatory interference theories (Burgess et al., 2007; Burgess, 2008) and other accounts (Pastoll et al., 2013; Wang et al., 2023). These frameworks often operate at distinct levels of description and timescales. Probabilistic models primarily address how sensory cues are integrated optimally; oscillatory accounts emphasize rhythmic and spike-timing mechanisms at the single-cell level; and continuous attractor or neural-field models focus on the timescale evolution of structured population activity.

Crucially, different theoretical perspectives may generate partially distinguishable predictions for certain experimental phenomena. Within a continuous-attractor framework, population activity is expected to exhibit low-dimensional continuous manifold structure, and local perturbations should typically produce smooth displacement along the manifold rather than discrete jumps between unrelated states. Such signatures have received support in both head-direction and grid-cell population recordings, although further large-scale measurements are required. By contrast, oscillatory or single-cell rhythm-based models attribute spatial patterning to underlying temporal structure, and may therefore predict distinct forms of instability or breakdown under rhythmic modulation. Purely probabilistic integration frameworks provide powerful accounts of cue weighting and bias, yet place fewer constraints on the geometric origin and stability of low-dimensional population structure. Accordingly, questions such as whether drift proceeds continuously along a representational axis, or whether remapping requires structural reconfiguration of the underlying dynamics, may yield different expectations across frameworks.

At the same time, we acknowledge that many observations remain compatible with multiple theoretical accounts. Boundary-induced distortions, remapping across environments, and some oscillation-manipulation experiments can be reproduced within attractor, oscillatory, or hybrid models under different assumptions. Viewing continuous attractors as a unifying language therefore does not imply exclusivity, but rather highlights that, when low-dimensional continuous geometry is present, diverse phenomena may be organized as modulations of a common dynamica structure.

### 4.7 Open problems

Although continuous-attractor and neural-field models provide a unifying account of many spatial coding phenomena, several challenges remain in elevating this framework to a predictive theory of closed-loop navigation.

#### 4.7.1 Mapping intrinsic structure to computational phenotype

While pattern-formation theory offers powerful tools, we still lack compact mappings–from biologically interpretable connectivity and nonlinearity parameters to pattern type, drift speed, and stability–in minimal two-dimensional neural-field models.

#### 4.7.2 Coordination of multiple modulatory mechanisms

Self-motion signals, sensory inputs, intrinsic circuit dynamics, environmental constraints, and attractor selection mechanisms jointly shape how population activity evolves during navigation. While each of these factors has been studied in isolation, a unified account of how their relative influence is dynamically coordinated remains incomplete. Evidence for near-optimal weighting of information sources in other neural systems (Kutschireiter et al., 2023; Jeffery et al., 2016) suggests that such coordination may reflect a general computational principle rather than a mechanism specific to navigation.

#### 4.7.3 From representation to behavior

While grid-cell and head-direction systems provide internal coordinate frameworks for space, closed-loop navigation requires additional mechanisms that transform these representations into action-relevant variables for planning, valuation, and sequence generation. How hippocampal-prefrontal circuits read out, manipulate, and control continuous spatial representations therefore remains a central open problem (McNaughton et al., 2006; Behrens et al., 2018; Farzanfar et al., 2023; Whittington et al., 2022; Bakermans et al., 2025). Importantly, conflict paradigms dissociating local and global reference frames indicate that the specific reference frame in which space is represented is often secondary to how representations are contextually selected, coordinated, and coupled to behavior. Rather than being resolved within a single attractor system, representational content, contextual modulation, and behavioral control appear to be distributed across interacting circuits (Knierim et al., 2014; Neunuebel et al., 2013; Save and Sargolini, 2017; Allison et al., 2023). These findings place strong constraints on how continuous attractor representations can be embedded within larger control architectures supporting flexible navigation.

#### 4.7.4 Temporal organization of attractor manifolds

While emphasizing the geometric and stability structure of continuous attractors, a natural extension concerns how low-dimensional attractor manifolds are organized and regulated in the temporal domain. Attractor-network models primarily describe the continuous representation of spatial variables and their evolution along a manifold, yet real navigation and memory processes are intrinsically embedded in time. Oscillatory rhythms, such as theta, may serve as temporal modulatory mechanisms within this framework, periodically adjusting network gain or effective coupling strength and thereby influencing both the stability of attractor states and the rate at which activity evolves along the manifold.

For example, against a background of theta oscillations, if the spatial phase on an attractor manifold advances continuously with the animal's movement while excitability is modulated rhythmically, neuronal firing phase may systematically precess relative to the oscillation, giving rise to spatiotemporal coding patterns of phase precession (Lisman and Jensen, 2013; Jensen and Lisman, 1996). In this scenario, continuous spatial trajectories are compressed and embedded within individual oscillatory cycles, forming temporally ordered spatial representations. From this perspective, attractor geometry provides the structural basis for spatial degrees of freedom, whereas oscillatory rhythms furnish the temporal framework. Their interaction may thus offer a dynamical mechanism by which location is embedded within a temporal sequence, potentially contributing to a unified spatiotemporal account of episodic memory (Eichenbaum, 2017) formation and retrieval.

## 5 Conclusion

Spatial navigation emerges from the controlled evolution of population activity on low-dimensional attractor manifolds, shaped by both intrinsic circuit structure and external constraints. Rather than treating drift, distortion, fragmentation, and remapping as disparate phenomena, a unified attractor-based perspective reveals them as consequences of how self-motion, sensory inputs, and environmental geometry modulate attractor dynamics at different levels. Distinguishing between additive drives, structural modulation, boundary constraints, and attractor selection provides a principled framework for organizing diverse experimental observations. More broadly, this perspective clarifies how continuous spatial representations can remain both stable and adaptable in the face of noise, uncertainty, and changing behavioral demands.
